# Recent Advances in Endolysin Engineering

**DOI:** 10.3390/antibiotics14121285

**Published:** 2025-12-18

**Authors:** Mackenzie Aitken, Gayan Abeysekera, Craig Billington, Renwick C. J. Dobson

**Affiliations:** 1 Biomolecular Interaction Centre, School of Biological Sciences, University of Canterbury, Christchurch 8041, New Zealand; mackenzie.aitken@pg.canterbury.ac.nz (M.A.); gayan.abeysekeraarachchige@pg.canterbury.ac.nz (G.A.); 2 Health Security Group, PHF Science, Christchurch 8041, New Zealand

**Keywords:** endolysin, multi-drug-resistant organism, antibiotic, antimicrobial resistance, protein engineering

## Abstract

Antimicrobial resistance threatens a new “dark age” in medical practice. Chronic antibiotic overuse has driven the rise in antimicrobial resistance and promoted the emergence of multidrug-resistant organisms. To address this problem, researchers have developed new approaches. Antimicrobials derived from bacteriophage, which are viruses that target bacteria, are promising candidates. Amongst these candidates, bacteriophage enzymes used in the viral replication cycle are of significant interest. Specifically, endolysins are used by bacteriophage to lyse the bacterial cell wall, leading to structural collapse and cell lysis. Researchers are increasingly applying these proteins externally to multidrug-resistant organisms as a novel antimicrobial treatment. Following this increased interest, many studies have presented protein engineering methods to further enhance the effectiveness of endolysins as antimicrobials. These methods include attachment of membrane-permeabilizing peptides, domain-swapping, and catalytic-site modification. Recent advances in all three fields have seen the implementation of tools like novel in silico design pipelines and library-based screening methods. This review summarizes these recent advances in the rapidly developing field of endolysin engineering and discusses potential future directions in this field.

## 1. Introduction

### 1.1. Antimicrobial Resistance

Antimicrobial resistance (AMR) is a serious issue in healthcare settings worldwide. Globally, AMR has directly resulted in an estimated 1.27 million deaths and contributed to another 5 million [1]. These numbers will likely rise over the coming decades, with global deaths from AMR predicted to reach ~10 million by 2050 [2,3]. AMR was estimated to cumulatively cost the global economy 693 billion USD in 2019 [4], rising to 100 trillion USD by 2050 [2].

The appearance of AMR is now understood to originate from excessive use of antibiotics [5]. High volumes of antibiotic use place selection pressure on bacteria, with those that are sensitive to the antibiotic dying and those with resistance phenotypes surviving and reproducing [6,7,8]. Experts refer to bacteria whose infections cannot be treated by multiple types of antibiotics as multidrug-resistant organisms (MDRO) [9]. These bacteria present an obvious threat to the global healthcare sector, where organisms such as *Pseudomonas aeruginosa*, *Acinetobacter baumannii*, *Staphylococcus aureus*, and *Escherichia coli* comprise most nosocomial infections [5,9,10,11,12]. MDRO infections are difficult to treat because the bacteria use a variety of resistance mechanisms [13] that are inherited through horizontal gene transfer from large community gene pools [5]. Furthermore, pharmaceutical company underinvestment has decreased the rate of development of new antibiotics as alternatives to conventional ineffective antibiotics [14,15]. Consequently, AMR threatens to send humanity back to the medical “dark ages”, where simple cuts may be life-threatening and advanced surgeries may become impossible [6]. Ideally, the solution to this looming crisis should both effectively eliminate problematic MDRO and limit, or even prevent, the development of resistance.

### 1.2. Bacteriophage and Their Use as Antimicrobial Therapeutics

Bacteriophage are viruses that infect bacteria, and are the most abundant biological entities on Earth [16]. First discovered at the end of the 19th century, bacteriophage were used effectively in the early 20th century to treat specific bacterial infections [17] by lysing the responsible bacterial strains. However, their use in Western medicine disappeared almost entirely upon the arrival of antibiotics, and remaining use was isolated to Eastern Europe [17,18]. This obsolescence likely occurred because of a combination of factors, including limited understanding of bacteriophage biology at the time and a lack of standardized methods for preparing clinical bacteriophage treatments [19]. The surge in MDRO has seen a shift in Western medicine’s focus back to these viruses. Interest in bacteriophage treatment was rekindled by promising results from experiments that were conducted with better designs and increased rigor compared with earlier experiments [20].

Interest in bacteriophage for medical treatment is grounded in their novel properties compared with the antibiotics traditionally used to treat infections. The main advantage of bacteriophage is their host specificity; bacteriophage often target certain species of bacteria [20,21]. This specificity limits the number of bacteria exposed to the treatment as a selection pressure and thus limits resistance evolution. Furthermore, this host specificity can leave other flora, such as those in the gut, untouched and protect patient gut health during treatment [22]. Other advantages of bacteriophage include their ability to “auto dose” via the natural bacteriophage replication cycle, which replenishes and increases the amount of the virus inside the patient, their relatively low production costs, and the speed of discovery and cultivation of specific bacteriophage for a host bacterium [21,22].

Despite its promise, bacteriophage-based therapy suffers from some disadvantages. First, bacteriophage contain genetic material and thus horizontal gene transfer between bacteria and bacteriophage is possible. Researchers have observed this in the temperate bacteriophage CTX, which delivers the gene encoding the toxin CtxAB to *Vibrio cholerae* to ultimately create bacterial strains that cause cholera symptoms [23]. Even if purely virulent bacteriophage are used, horizontal transfer of genetic elements to bacteria can occur via transduction [24]. Second, by accessing the clustered regularly interspaced short palindromic repeats-associated system, bacteria can form an adaptive immune response against previously encountered bacteriophage [25]. This system allows for cleavage of the phage genetic material, preventing replication and subsequent bacterial lysis [26]. Third, regulatory hurdles regarding bacteriophage-based therapies currently affect their ability to be distributed in clinical settings. Bacteriophage are deemed an “active treatment” because of their self-replicating ability and thus are in a completely different class to traditional passive antibiotics [27]. The above issues have halted progression of legislation that is needed to facilitate clinical use of bacteriophage.

In summary, bacteriophage show great potential for use as an alternative antimicrobial treatment, but there are some issues hindering their full clinical use. Exploiting certain aspects of bacteriophage machinery as antimicrobial tools may help to address these issues.

### 1.3. Endolysins and the Bacterial Cell Wall

Endolysins are protein enzymes that show promise for bacteriophage-based antimicrobial treatment. First reported in 1974, endolysins are peptidoglycan hydrolases that are expressed during the late stages of bacteriophage replication and cleave the cell wall of the host bacteria from within, causing cell lysis [28,29,30,31]. This cell lysis releases progeny phage to initiate many new bacteriophage infection cycles. Endolysins are distinct from lytic enzymes because they facilitate cell lysis for progeny phage release rather than inducing local lysis to facilitate bacteriophage genome injection [28]. Many studies have reported on the potential use of endolysins as antimicrobials when applied exogenously to bacteria [32,33,34]. The effectiveness of endolysins as novel antimicrobials stems from the advantages they have over traditional antibiotics.

To date, no studies have observed the development of bacterial resistance to phage-derived endolysins [28,35], even when they are used at identical concentrations and doses as other antibiotics that lead to resistance [36]. This lack of resistance evolution is thought to occur because endolysins target such a critical structural molecule (peptidoglycan) in the host bacteria. Researchers have speculated as to whether this is a product of an evolutionary “assurance policy”, with bacteriophage endolysins evolving to target this critical molecule to ensure that resistance is rare and that progeny virus release will always occur [37]. Additionally, because endolysins only target the cell surface, they avoid bacterial resistance mechanisms that are frequently mediated inside the cell [30]. Another factor limiting resistance development is that endolysins typically display a narrow host range [28,30]. With bacteria, broad-spectrum antibiotic use can result in evolution of resistance phenotypes in neighboring commensal bacteria because of genotype sharing through horizontal gene transfer [30]. This evolution of resistance phenotypes leads to the emergence of antibiotic-resistant bacterial strains. By contrast, endolysins avoid these issues because they have relatively limited host ranges and are active only with particular strains of a species [35,38] or other Gram-negative species [32,39] in addition to their Gram-negative host target. The activity of endolysins with Gram-negative species other than their host target is attributed to conservation of the peptidoglycan structure and composition in Gram-negative organisms [40].

Endolysins have advantages over bacteriophage for prospective clinical treatment. First, unlike bacteriophage, endolysins are classified as “passive pharmaceuticals” rather than “active pharmaceuticals”, which means their application is not hindered by the same regulations [27]. Furthermore, because endolysins contain no genetic information, bacteria cannot use the clustered regularly interspaced short palindromic repeats-associated system to develop resistance. This means they also cannot inherit new genes via bacteriophage transduction, as mentioned above. Because of these differences, the regulatory hurdles for endolysins are lower than those for bacteriophage.

**Figure 1:**
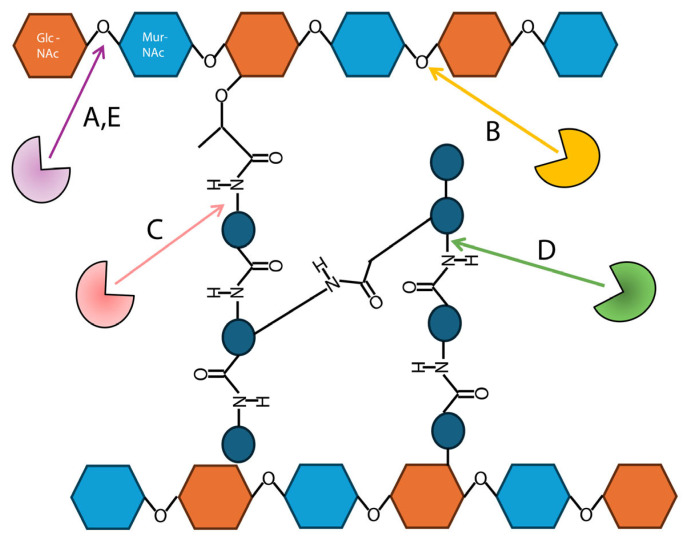
The basic structure of the peptidoglycan macromolecule and the various catalytic targets of endolysin enzymatically active domains. Repeating units of *N*-acetylglucosamine (Glc-NAc) and *N*-acetylmuramic acid (Mur-NAc) are connected by peptide bridges. Target sites (indicated by arrows) of different types of endolysins: A, *N*-acetyl-β-d-muramidases; B, *N*-acetyl-glucosaminyl-β-d-glucosaminidases; C, *N*-acetylmuramoyl-l-alanine amidases; D, endopeptidases; and E, lytic transglycosylases (these enzymes differ from *N*-acetyl-β-d-muramidases and do not use a water molecule for catalysis).

Endolysins specifically target the major structural molecule of the cell wall, peptidoglycan, to initiate cleavage. This composite molecule contains alternating *N*-acetylglucosamine and *N*-acetylmuramic acid units linked by β-1,4 bonds to form long biopolymers, and stem peptides that link these sugar polymers in parallel [28,30] (Figure 1). There are five unique classes of endolysins that are characterized by where bond cleavage occurs in the peptidoglycan biomolecule during cell wall lysis [28,30] (Figure 1). Endolysins classed as *N*-acetyl-β-d-muramidases and lytic transglycosylases target the *N*-acetylmuramoyl-β-1,4-*N*-acetylglucosamine bond between the *N*-acetylglucosamine and *N*-acetylmuramic acid disaccharide of the peptidoglycan sugar backbone, while those classed as *N*-acetyl-glucosaminyl-β-d-glucosaminidases target the *N*-acetylglucosaminyl-β-1,4-*N*-acetylmuramine bond [30,37,41]. The sugar backbone of peptidoglycan is not the only target of endolysin cell wall lysis. Endolysins classed as *N*-acetylmuramoyl-l-alanine amidases cleave the amino acid peptide connecting the l-alanine of the peptide to the sugar backbone at various locations (depending on the host species) [30,37,41]. Even though different endolysins target different bonds in peptidoglycan, they lead to the same cell lysis effect. Another factor affecting endolysin cell wall lysis is that the location of peptidoglycan in the cell wall differs between Gram-positive bacteria, which have a thick layer of peptidoglycan external to their cell membrane [28], and Gram-negative bacteria, which have a thinner layer of peptidoglycan encased by the inner and outer membrane [39,42,43]. Between both Gram types, all five endolysin classes lyse peptidoglycan the same. Because of their outer membrane, Gram-negative bacteria are notoriously difficult to treat with antibiotics [44] and endolysins [37]. Mirroring these differences in cell wall peptidoglycan, endolysins from bacteriophage that target Gram-positive and Gram-negative bacteria have distinguishable differences in their architecture.

### 1.4. Endolysin Structure and Catalysis

Endolysin structures can contain two types of domains: the cell wall-binding domain (CBD), and the enzymatically active domain (EAD). The CBD is responsible for recognizing and binding specific moieties on the host peptidoglycan, often carbohydrates, for efficient peptidoglycan breakdown [37]. Whereas the CBD is not always required for successful bacterial cell lysis [45,46,47], the EAD is responsible for an endolysin’s ability to cleave the cell wall. The CBD often targets specific strains or closely related species [30]. Scientists have used this specific targeting to repurpose CBDs as inexpensive antibodies and tested them for use in the food industry [48].

**Figure 2:**
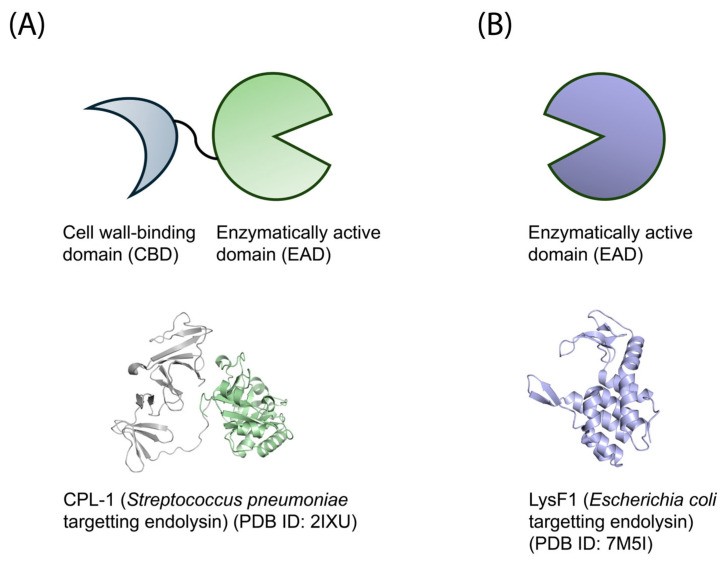
The typical domain architecture of Gram-positive and Gram-negative targeting endolysins. (**A**) Endolysins that target Gram-positive bacteria typically have both a CBD and EAD. (**B**) Endolysins that target Gram-negative bacteria tend to only have an EAD.

The specific domains present in an endolysin depend on whether it targets Gram-positive or Gram-negative bacteria (Figure 2). Endolysins that target Gram-positive bacteria contain both a CBD and an EAD. In this case, the CBD binds to specific ligands common to the thick peptidoglycan layer that is characteristic of Gram-positive organisms. These ligands can include carbohydrates on the cell wall surface [49] and choline molecules bound to glycan strands [50]. Conversely, most endolysins that target Gram-negative bacteria only contain an EAD. This lack of a CBD is likely because its target, the peptidoglycan layer, is sandwiched between two lipid bilayers and is notably lacking in surface epitopes [30].

As well as shielding the peptidoglycan layer, the lipid bilayer of Gram-negative bacteria makes it difficult to apply endolysins that target Gram-positive bacteria to kill Gram-negative bacteria [32,39]. Because of the modular, dual-domain nature of Gram-positive targeting endolysins, researchers have experimented with domain swapping/domain addition to alter their selectivity and effectiveness against novel bacterial targets [51,52]. One study even created a potent chimeric enzyme by combining the EAD of a phage endolysin with the CBD of a host bacterium autolysin (used natively to recycle peptidoglycan as the cell grows) [53]. It is clear that this structural architecture is promising for future engineering of endolysin therapeutics. However, it is not the only aspect of these proteins that can be manipulated to enhance their lytic activity.

### 1.5. Endolysin Engineering

Endolysins show excellent potential as antimicrobials when applied externally to bacteria [32,54,55,56]. In vivo studies have shown bactericidal effects when mice infected with various *Streptococcus* species are treated with endolysins [57,58,59]. An endolysin’s ability to kill bacteria when applied externally can be enhanced via multiple mechanisms. Modifications can even induce potent antimicrobial activity against MDRO. There are currently three main engineering strategies for enhancing an endolysin’s ability to kill bacteria:

- Fusion of a cell-membrane permeabilizing peptide to Gram-negative targeting endolysins [29,60,61].
- Swapping or addition of endolysin domains [51,52,53].
- Modification of endolysin catalytic sites [62].

Each strategy represents a distinct way of optimizing endolysin therapeutics to kill the targeted bacterium more efficiently. Importantly, these strategies relate to modifying endolysins to enhance lytic activity, rather than optimizing parameters such as bioavailability, stability, and delivery. These different engineering strategies have recently evolved in innovative ways that bode well for the future of endolysin engineering. This review summarizes the latest endolysin engineering efforts for each of these three strategies and critically evaluates knowledge gaps and future engineering trends.

## 2. Fusion of Membrane Permeabilizing Peptides to Gram-Negative Targeting Endolysins

### 2.1. Overcoming the Outer Membrane Barrier

**Figure 3:**
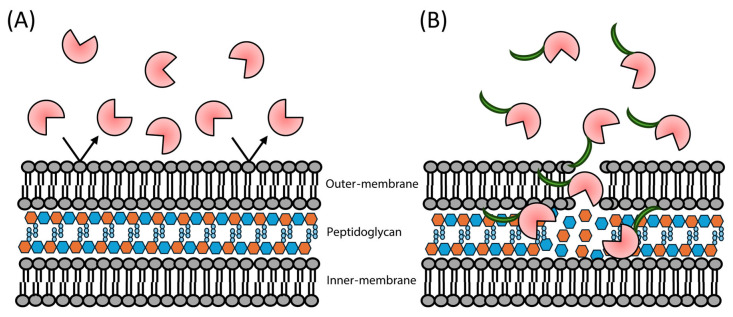
Visual representation of how Artilysin engineering can enhance peptidoglycan lysis. (**A**) Many endolysins lack a native mechanism of permeabilizing the outer membrane, preventing them from accessing the peptidoglycan. (**B**) Fusion of membrane-permeabilizing peptides to these endolysins allows them to bypass the bacterial outer membrane, increasing their effectiveness as antimicrobials.

A significant hurdle when attempting to treat Gram-negative bacteria with endolysins is the outer membrane [63]. This structure serves as an innate resistance mechanism against many antimicrobial treatments, preventing their entry through to the cell wall in the case of endolysin, or the cytoplasm in the case of small molecule antimicrobials [64]. Only a small number of endolysins exhibit native membrane-permeabilization abilities that enable them to cleave the cell wall when externally applied to Gram-negative bacteria [32,65]. The mechanism of this permeabilization is currently unknown in all cases. Grafting with membrane-permeabilizing peptides to enhance the endolysin’s cell-killing ability has attracted attention. This method allows these proteins to access Gram-negative peptidoglycan, which mediates cell-wall cleavage and subsequent cell lysis. First hypothesized at the start of the 2010s [66], this idea was executed shortly afterwards against *P. aeruginosa* with great effect [29,60,67]. The resulting engineered endolysins are referred to as “Artilysins” (Figure 3).

### 2.2. Artilysin Engineering to Enhance Endolysin Cell Killing

Several studies have reported important advances in Artilysin engineering, and are summarized in Table 1.

In 2021, Wang et al. reported the fusion of a previously characterized *P. aeruginosa* endolysin, LysPA26, with a sheep myeloid 29 amino acid peptide (SMAP-29) to enhance LysPA26 cell killing. The study was analogous to one conducted in 2016 with the *P. aeruginosa* endolysin KZ144 [60]; however, LysPA26 was chosen because of its wider host range [68]. Additionally, Wang et al. investigated whether the length of the linker sequence connecting the antimicrobial peptide to the endolysin affected the cell-killing ability. Three protein constructs were created that contained LysPA26 and SMAP-29 but had linker sequences of different lengths (3, 9, or 15 amino acids). Once purified, these constructs were incubated with the multidrug-resistant strain of *P. aeruginosa*, PAO1. Ultimately, the construct containing LysPA26 and SMAP-29 with a linker of three amino acids showed a far larger increase in cell killing ability than constructs with other linker lengths or LysPA26 alone. The cell-killing effect was rapid, dose-dependent, and also observed in some other Gram-negative species. The effect of linker length on cell-killing ability was correlated with changes induced in the endolysin secondary structure by the grafted antimicrobial peptides. Notably, linker lengths of 9 and 15 amino acids resulted in lower alpha helix percentages (54.25% and 40.09%, respectively) than wildtype LysPA26 (91.22%). This study highlights that domain fusion linker length is an important factor in Artilysin activity.

In another study, Nie et al. engineered an Artilysin entirely in silico and then experimentally showed it inhibited *Salmonella* [69]. Four candidate endolysins were selected from 150 present in the genomes of various *Salmonella* bacteriophages. Antimicrobial peptides were designed by taking the LeuA peptide, which has low antimicrobial ability, and optimizing its charge and hydrophobicity using bioinformatic programs. The charge and hydrophobicity are critical for antibacterial activity [72]. To ensure the Artilysins were able to bind to key biomolecules in the host bacteria, in silico molecular docking experiments were performed to test the binding affinities of the proteins to lipopolysaccharides and peptidoglycan. All four Artilysins were predicted to have high affinity to both lipopolysaccharides and peptidoglycan. In particular, the affinities for the phosphate moiety and peptidoglycan were related to their abilities to permeabilize the outer membrane and cleave the host bacteria peptidoglycan, respectively. All four proteins were successfully expressed and purified, and used in subsequent in vitro bactericidal assays, where they showed inhibitory effects on various *Salmonella* serotypes exclusive to this organism only. The cell-killing ability was not seen when tested on any other bacterial species. Importantly, membrane permeabilization was directly observed as a change in the absorption wavelengths at 350/429 nm upon addition of the Artilysins to *Salmonella* cultures. *N*-Phenylnaphthylamine (NPN)- and 3,3′-dipropylthiadicarbocyanine iodide [DiSC3(5)]-based assays were used to quantify outer and inner membrane permeabilization, respectively. These dyes fluoresce in different environments. NPN is weakly fluorescent in aqueous environments. When it enters bacterial cells, its fluorescence increases because the cytoplasm is relatively hydrophobic compared with the external environment. For DiSC3(5), when the dye is absorbed by bacteria, its fluorescence is self-quenched because of the proximity of dye molecules. Cell permeabilization results in release of DiSC3(5), a decrease in its concentration within the bacteria, and an increase in its fluorescence. Overall, this work highlights how bioinformatics can be leveraged to engineer enhanced antimicrobial peptides for use in Artilysins. In future, comparing the cell-killing abilities of these Artilysins against those of the same endolysins with the original LeuA peptide will help to quantify the improvement in activity.

In 2023, Ning et al. created Artilysins by fusing the endolysin Lys with 21 different outer-membrane-destabilizing peptides [70], and then tested them against *Vibrio parahaemolyticus*. They investigated whether a library-based approach generated Artilysins that were effective against *V. parahaemolyticus*. Prior to this, Ning et al. showed that the cell-killing and membrane-permeability abilities of Lys were enhanced by grafting with three cationic peptides of different sizes [73]. In the 2023 study, a larger collection of peptides were fused to either the N- or C-terminal of the endolysin, with high acceptability predicted via Ramachandran plots and programs that determine the quality of tertiary structure. Like in the study by Nie et al. [69]. Ning et al. used molecular docking. They found that most of the Artilysin constructs had high binding energies to lipopolysaccharide lipid A and similar binding energies to peptidoglycan, relative to the parent endolysin Lys. The Artilysin constructs with the best predicted binding energies were then transformed, expressed, and purified; however, only a handful of constructs were successfully expressed and purified. When tested in bactericidal assays, these proteins exhibited minimal inhibitory concentrations of 50–800 μg/mL. Additionally, these Artilysins killed all *V. parahaemolyticus* strains tested and various other Gram-negative species. Compared with previous studies using a single type of peptide, this study demonstrates the promise of attaching a variety of previously characterized antimicrobial peptides to the same endolysin.

Usually, Artilysin engineering is a time-intensive process that ultimately produces individual designs. Zeng et al. used Artilysin recombinant libraries to expedite creation of new Artilysins [71]. A high-throughput method for screening and expressing novel Artilysins was created using a composite library of 38 antimicrobial peptides and 8 endolysins. All combinations were then cloned and plated. Colonies were evaluated using growth inhibition and turbidity reduction assays against *P. aeruginosa* PAO1. The three colonies with the highest growth inhibition were selected for protein purification and further characterization. These Artilysins showed effective cell-killing abilities (4.93–6.75 log_10_ colony-forming unit reductions), were highly refractive to resistance development, showed broad-spectrum antimicrobial activity against both Gram-positive and Gram-negative bacteria, and demonstrated independent membrane permeabilization ability via the *N*-phenylnaphthylamine and DiSC3(5)-based assays. Crucially, the effectiveness of these Artilysins as antimicrobials was greatly improved when compared with their parent endolysins. This study shows that high-throughput expression and screening of many different Artilysin constructs is effective for selecting viable Artilysin candidates from hundreds of options.

### 2.3. Future Directions for Artilysin Research

Artilysin engineering has shifted from using a single endolysin and a handful of antimicrobial peptides, or vice versa, to library-based screening methods for high-throughput workflows. Although the Artilysins concept is robust, an improved selection method is required to ensure it does not take years to develop a handful of engineered proteins. Future work should build upon the high-throughput library method using a wider range of both endolysins and antimicrobial peptides to develop a larger catalog of Artilysins to fight AMR. Further optimization of these screened Artilysins via in silico techniques to enhance their cell-killing abilities could improve their function and minimize resource waste on optimizing less-efficient Artilysins.

Although many studies have demonstrated that Artilysins show enhanced cell-killing abilities upon grafting with an antimicrobial peptide, the actual mechanism of permeabilization is still poorly understood. Further investigations into how these peptides realize membrane permeabilization will aid optimization of in silico design techniques, including training artificial intelligence (AI) models. Combining membrane permeabilization assays with protein mutagenesis will help to elucidate which residues are important for function and could serve as a framework for modeling the mechanisms.

## 3. Swapping or Addition of Endolysin Domains

### 3.1. Overview of Endolysin Domain Swapping

**Figure 4:**
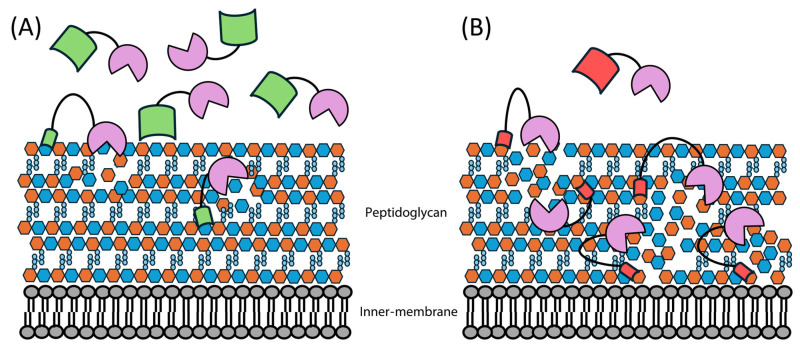
Overview of how domain swapping can enhance endolysin cell-killing ability. (**A**) Endolysins (mauve) containing their original CBD (green) still exhibit cell-killing ability. (**B**) Endolysins with their CBD swapped for another (red) show more effective cell-killing relative to the parent endolysin, due to their increased cell wall binding ability.

Domain-swapping engineering involves taking a functional domain from one endolysin and placing it in another endolysin, for example, swapping a domain that targets one bacterial host with one that targets another. This can change the host selectivity whilst retaining the catalytic ability or enhancing the cell-killing ability relative to the parent endolysin (Figure 4). Gram-positive endolysins are well suited to this approach because they typically have modular domain architecture, with both a CBD and an EAD. Most research in this field has focused on these endolysins; however, some recent novel approaches have expanded upon the basic concept of shuffling domains and comparing cell-killing ability (Table 2).

### 3.2. Domain Swapping Effects on Antimicrobial Activity

Domain shuffling using endolysin libraries is one of the most frequently reported new methods. Recent experiments have used libraries of *S. aureus* targeting endolysins to show that a library-based approach can effectively enhance activity and cell-killing ability for MDRO previously untouched by the donor endolysins. Lee et al. constructed a library using 12 CBDs and 22 EADs from 12 native *Staphylococcus* endolysins and then fused these domains in a variety of combinations to create 600 chimeric constructs [52]. The chimeric constructs were experimentally produced via a two vector endolysin/spanin expression system and screened using lysis zones on bacterial lawns. The same library-based chimeric endolysin expression system was used by Son et al. to generate 480 clones from only four endolysins [40]. Importantly, two different libraries were generated: constructs with one CBD and one EAD, and constructs with one CBD and two EADs. In combination with a high-throughput screening assay, both studies quickly identified chimeric endolysins with the best cell-killing abilities for further characterization. Importantly, chimeric constructs are often more potent than their original donor endolysins. Consequently, as shown by Lee et al., their cell-killing ability is enhanced against various clinical *S. aureus* isolates. As an example, enhanced cell killing was observed for a *S. aureus* strain isolated from patients with bacteremia at a South Korean hospital [52]. Alongside this work, another study conducted similar domain-swapping experiments using small endolysin domain libraries [77] and reported successful increases in cell-killing ability relative to the parent endolysin.

Aside from library-based domain-swapping approaches, work focusing on single endolysins demonstrates that domain swapping improves both cell-killing ability and practical application. Wang et al. showed that combining the cysteine, histidine-dependent amidohydrolase/peptidase (CHAP) domain and cell wall-binding domain from separate *Streptococcus* targeting endolysins (Ply2741 and PlyV12, respectively) produced a chimeric endolysin (Cly2v) with greater cell-killing ability against various *Streptococcus* species than Ply2741 [74]. Interestingly, Cly2v showed greater killing against *Streptococcus* species that induce bovine mastitis in dairy cows. It also greatly reduced streptococci-induced mastitis in mouse models by lowering both their bacterial burden and inflammation. Thus, Cly2v shows great potential for practical application in the field. Because Cly2v has the same EAD (CHAPS) as its parent endolysin Ply2741 but a greater cell-killing ability, the implication is that the new CBD increases the binding affinity of Cly2v to the peptidoglycan of the *Streptococcus* species. These results demonstrate how domain swapping by changing only one CBD can dramatically change the effectiveness of an endolysin. Similarly, Saber et al. demonstrated that adding a *S. aureus*-targeting CBD to an endolysin with only a single catalytic domain (LYZ2) led to a new chimeric protein with a 64-fold reduction in both the minimum inhibitory concentration and the minimum bactericidal concentration compared with the parent [51]. Again, the importance of the CBD is readily apparent. According to this research, the scope of engineering opportunities with Gram-positive endolysins via domain-swapping methods seems limitless.

Exchanging or adding domains is not the only way to enhance an endolysin’s effectiveness as an antimicrobial. Agún et al. recently showed that deletion of an amidase domain from the *S. aureus*-targeting endolysin LysRODI increased its cell-killing ability against various isolates. This change was observed at a range of incubation temperatures, and the chimeric endolysin was more effective than its parent at killing bacteria during the cheese production process [75]. Although previous research has explored the concept of domain deletion [78], testing of the effectiveness of chimeric endolysins under various in vitro and in vivo conditions is needed to demonstrate the benefits of domain-swapping engineering for enhancing the effectiveness of a parent endolysin.

### 3.3. Domain Replacement with Other Bacteriophage Proteins

Until recently, reports on domain swapping were limited to Gram-positive targeting endolysins. This was likely because there were very few characterized Gram-negative endolysins with two domains. However, this method can now be applied because of the discovery of Gram-negative targeting endolysins that contain multiple domains and have structures resembling those of Gram-positive endolysins [32,79,80]. For example, Warring et al. used a DNA shuffling technique to create chimeric endolysins from Gram-negative endolysin EADs and various other phage-encoded proteins, including holins, tail proteins, and receptor binding proteins [76]. Among the approximately 2200 chimeras developed in this study, 409 were selected from medium-level screening for antimicrobial activity. A lead candidate was identified that contained the EAD of an endolysin homologous to the *P. aeruginosa* targeting GP144 and the lipase domain from a bacteriophage with siphovirus morphology. This candidate exhibited exceptional cell-killing ability when combined with small amounts of citric acid or ethylenediaminetetraacetic acid and killed a narrow range of *Pseudomonas syringae* strains. However, *Pseudomonas aeruginosa* and Gram-positive *S. aureus* were not affected by the engineered candidate, regardless of the protein or additive concentration. The domains required linkage to elicit optimal cell-killing and, interestingly, positioning of the EAD at the N-terminus and the lipase at the C-terminus gave greater cell-killing ability than the inverse combination. This study demonstrates that chimeric engineering involving Gram-negative endolysins (in this case, the enzymatically active domain from an endolysin homologous to GP144) is possible. Furthermore, combining endolysin domains with other phage proteins is a promising avenue for endolysin engineering.

### 3.4. Future Directions for Endolysin Domain-Swapping Engineering

The domain-swapping engineering approach has clearly evolved from its early days of simply exchanging endolysin CBDs and assessing cell-killing ability [81,82]. Nonetheless, in most cases, basic domain-swapping sees chimeric endolysins with increases in cell-killing ability both in vitro and in vivo. Recent improvements in engineering have solidified its power as a tool for improving endolysin effectiveness. Like Artilysin engineering, domain-swapping shows potential for reviving endolysins that have sub-optimal cell-killing abilities or undesired host ranges.

Interestingly, many studies that did not use a library-based screening approach focused on swapping the CBD only. Swapping of the CBD and EAD with domains from other endolysins is possible, provided there are no expression or solubility issues. Future studies could investigate whether swapping the EAD while retaining the CBD (the inverse of the experiment conducted by Wang et al. [74]) enhances cell killing. For instance, an endolysin catalytic domain may see better activity against the peptidoglycan of another species. To date, library-based screening studies of various EAD and CBD combinations have not investigated this.

Few studies have reported on domain swapping with Gram-negative targeting endolysins, largely because modular domains are rare in this class. Future research could explore domain swapping with these types of endolysins to enhance their selectivity and cell-killing ability. Although Warring et al. [76] investigated chimeric endolysins with other bacteriophage proteins, no studies have reported on swapping the endolysin CBD between Gram-negative endolysins and whether this could increase the cell-killing ability. Recent advances in Gram-positive domain swapping, and the recent Gram-negative endolysin/phage protein chimera [76], will likely pave the way for using similar techniques on Gram-negative dual domain counterparts. This ability to mix and match endolysin domains will expand the range of Gram-positive and Gram-negative endolysins available to combat pathogenic bacteria.

## 4. Modification of Endolysin Catalytic Sites

### 4.1. Endolysin Catalytic-Site Engineering in Clinical Use

Accessing the peptidoglycan layer of bacteria exogenously with endolysins often requires advanced protein engineering techniques and precise candidate selection strategies [83,84,85]. However, if the catalytic domain of an engineered endolysin targets a bond that is prone to structural variation, such as in cross-linked peptides, or acts non-specifically against beneficial bacterial strains, any investment in developing such an enzyme may be wasted. Therefore, catalytic-domain engineering is as important as other aspects of endolysin design. Some recent examples of this engineering are summarized in Table 3.

The potential of the catalytic domain is influenced by the composition of the catalytic site and the modularity of the endolysin, which together determine its specificity, efficiency, resistance profile, and biofilm-disrupting ability [86,87]. The PlyC endolysin (CHAP + lysozyme domain) selectively targets *Streptococcus* species [88], while LysK endolysin (amidase + CHAP) effectively lyses *Staphylococcus* [89]. Highly efficient endolysins, such as Cpl-1 and LysEF-P10, enable rapid bacterial clearance in vivo [90,91]. Active sites targeting conserved peptidoglycan bonds, like those in Exebacase lysin [92] and PlySs2 endolysin [93], remain effective against multidrug-resistant strains. Finally, domains capable of cleaving cross-linked peptides, including CHAP and amidase active sites in LysK, LysH5, and engineered P128, disrupt biofilms on medical devices and assist in decolonization strategies. Although most endolysins have CBDs that confer specificity for binding to peptidoglycan, some data suggest that they have a synergistic effect on the catalytic site [93]. Building upon these insights, mutagenesis, active site modifications, and modular strategies for catalytic-site engineering show potential to further enhance catalytic efficiency, broaden specificity, and optimize therapeutic performance.

### 4.2. Mutagenesis and Active-Site Modifications

Although mutagenesis and active-site modifications for endolysin engineering are conceptually simple, they are highly challenging in practice because of the complexity of the active site, protein folding, the substrate, and environmental factors. Despite these challenges, Mayer et al. [86] used mutagenesis to fine-tune the species specificity in the catalytic domain of the endolysin CD27L. Naturally, CD27L induces cell lysis in the pathogen *Clostridium difficile.* The researchers mutated Leu98 in CD27L to a Trp residue, a substitution previously observed in a bacteriophage endolysin targeting *Listeria monocytogenes*, to successfully confer bactericidal activity against *L. monocytogenes* [86]. In another study, mutagenesis of the noncatalytic gating residue H37A in the catalytic cavity in T7 amidase increased the enzymatic activity by approximately 35% and optimized the balance between stability and activity [94]. This finding highlights how substrate-access residues in the catalytic domain can strongly influence enzymatic efficiency. In further research, Love et al. demonstrated that the activity of the core variant Glu88Met increased to 123% of the wild-type level, while also enhancing the overall stability of the protein [39].

Low et al. [95] used a different approach that involved altering the net charge of the catalytic domain (Zn^2+^-dependent amidase) to investigate its influence on protein activity. They proposed that modifying the net charge of the catalytic domain offered a straightforward strategy to refine or expand the host range of lysins [95]. However, a more recent study (2019) examined the effect of net charge on the CHAP domain of the PlyC endolysin and found that none of the mutants were more active than the wild-type CHAP. Thus, the authors suggested that charge should remain an important consideration in future engineering efforts, but not the sole focus [96].

**Figure 5:**
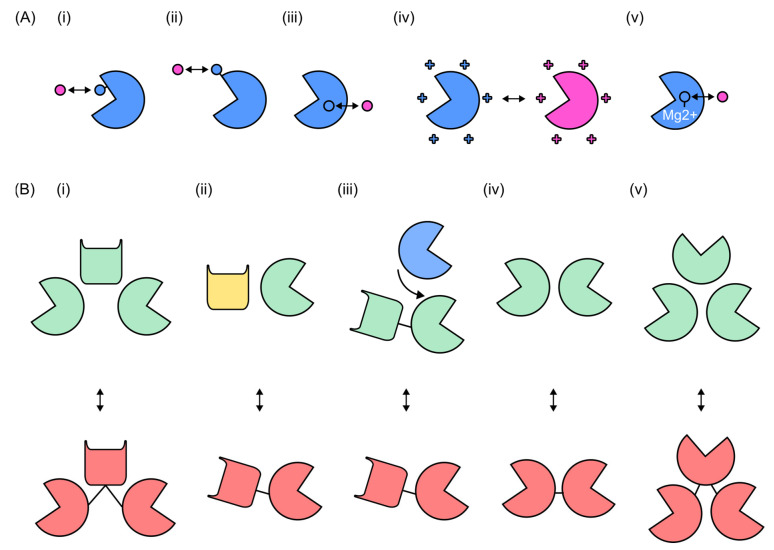
Schematic representation of catalytic endolysin engineering strategies and opportunities. (**A**) Mutagenesis and active-site modifications: (**i**) alteration of the catalytic site, (**ii**) modification of noncatalytic gating residues, (**iii**) modification of core residues, (**iv**) modulation of net surface charge, and (**v**) engineering of metal-binding pockets. The native enzyme state and engineered or altered residues are shown in blue and pink, respectively. (**B**) Modular strategies in catalytic endolysin engineering: (**i**) exploitation of intramolecular synergy, (**ii**) construction of chimeric catalytic endolysins, (**iii**) development of endolysin–bacteriocin hybrid enzymes, (**iv**) design of dual catalytic domain lysins, and (**v**) generation of triple-acting multifunctional lysins. Native configurations are shown in green, blue, and yellow, and engineered variants exhibiting enhanced or novel properties are shown in dark pink. Arrows indicate engineered variants.

Another charge-related factor in endolysin activity is the presence of metal ions. Several studies have reported a loss of or reduction in activity when attempting to engineer the metal-binding pocket (Zn^2+^ or Ca^2+^), with none observing an increase in activity. These results indicate that the metal-binding pocket may offer fewer engineering opportunities than the catalytic site, active site residues, or noncatalytic gating residues [91,97,98,99] (Figure 5A).

### 4.3. Modular Strategies in Endolysin Catalytic-Site Engineering

Compared with mutagenesis and active site modifications, domain-level engineering of endolysins offers greater flexibility and opportunities. As discussed earlier, the modular organization of Gram-positive endolysins underpins their versatility and makes them prime candidates for endolysin catalytic-site engineering. Modularity enables strategies such as rational domain fusion, chimeric constructs, and dual catalytic configurations, all of which expand functionality beyond that of the parent enzyme.

Naturally occurring multidomain lysins demonstrate intramolecular synergy between catalytic and noncatalytic domains. This synergy provides a valuable strategy for endolysin catalytic-site engineering. For example, PlySK1249 is a bifunctional lysin containing a CHAP domain and an amidase domain linked by a central CBD. Research has shown that these catalytic domains are likely to act cooperatively to optimize bacterial lysis even though their individual activities are comparatively weak. This study also indicated that because of this synergistic behavior, domains lacking direct lytic activity, such as CBDs or CBD-like domains, should not be disregarded [87]. Intramolecular synergy may arise from the specificity of the CBD toward cell wall components; however, other researchers have shown that truncation of the C-terminal SH3b cell-binding domain in LysK endolysin results in higher activity than the wild type [89]. Another example is the truncated Ecd18980CD endolysin, where the position of peptide bridge-cleavage sites relative to the enzyme’s active center limits access to certain substrates [100]. This leads to species-specific lytic activity, even though Ecd18980CD has a CBD-like domain [100]. These results suggest that such synergy may not rely solely on the CBD specificity for cell wall components. To date, intramolecular synergy has not been thoroughly investigated.

Engineered catalytic chimeras can enhance both specificity and antibacterial potency. One such example is Ply187AN-KSH3b, which replaces the two catalytic domains of LysK endolysin with the EAD of Ply187 endolysin. Compared with full-length Ply187, the truncated variant Ply187AN, and the well-characterized high-activity LysK endolysin, the chimeric Ply187AN-KSH3b exhibits stronger antimicrobial activity and broader and more potent staphylococcal killing [82]. More recently, the same highly active catalytic domain was used to replace phiNM3 endolysin containing a non-SH3b CBD, generating a chimeric lysin called ClyH. This construct successfully killed methicillin-resistant *S. aureus* (MRSA) strains. The researchers concluded that ClyH demonstrated strong antimicrobial activity against all tested clinical MRSA isolates while exhibiting improved properties compared with other lysins [101]. Comparable results were observed for the engineered Lys109 endolysin, which was derived from LysSA12 and LysSA97 containing CHAP, amidase, and CBDs. In this construct, the CHAP domain of LysSA12 was fused to LysSA97, producing Lys109, which displayed much stronger lytic activity against staphylococcal strains than its parent endolysins. The researchers hypothesized that the enhanced antibacterial activity of the chimeric endolysin resulted from its increased binding affinity to target bacteria or the improved effectiveness of the engineered CHAP domain [77].

Endolysin–bacteriocin chimeras further exploit this architecture, as demonstrated by the Ply2638-derived constructs SA.100 and XZ.700. In these constructs, the weak M23 endopeptidase of Ply2638 was replaced with the catalytic domain of lysostaphin and the N-terminal linker was optimized, resulting in a significant boost in activity [102]. Idelevich et al. proposed a two-domain structure of HY-133 consisting of an N-terminal EAD derived from the CHAPK domain of endolysin LysK and a C-terminal CBD originating from the SH3b domain of lysostaphin, with the domains connected by a linker of 17 amino acids. This engineered configuration expanded the spectrum of HY-133 activity to include MRSA [103].

Uniquely, dual catalytic-domain lysins can simultaneously hydrolyze distinct bonds within the peptidoglycan. This reduces the likelihood of resistance and ensures activity across diverse physiological conditions, as highlighted by Rodríguez-Rubio et al. [104]. There are several natural and engineered examples to support this concept. However, in most dual-EAD staphylococcal endolysins, lytic activity is predominantly driven by the N-terminal CHAP endopeptidase domain, while the amidase domain remains largely inactive when applied externally [105,106,107]. This observation suggests that replacing inactive or inefficient domains presents a catalytic-site engineering opportunity to design potent chimeric variants capable of effectively targeting and killing bacterial pathogens. The pneumococcal chimeric lysin Cpl-711, which combines the EAD (muramidase) domain of Cpl-7 with the amidase domain of Cpl-1, demonstrates superior killing activity against *Streptococcus pneumoniae* and enhanced resilience to resistance compared with its parent enzymes [108]. Another example is the naturally occurring PlySK1249, a multidomain lysin containing amidase and CHAP catalytic modules coordinated by a LysM binding domain, which exhibits strong intramolecular synergy that boosts lytic efficiency [87]. Similarly, the multimeric streptococcal lysin PlyC incorporates both CHAP and glycosidase activities and stands out as one of the most potent lysins described to date [88,109]. LysSYL, which combines a CHAP domain with an amidase, displays broad-spectrum lytic activity against *Staphylococcus* species and effectively eradicates *S. aureus* biofilms [110]. Lys84 has a similar dual-domain configuration and lytic profile, underscoring the advantages obtained when combining multiple catalytic mechanisms [111]. Researchers observed comparable outcomes with the chimeric Lys109, which they engineered using two naturally occurring dual endolysins to enhance activity against diverse Gram-positive species, including biofilms [77]. Expanding beyond dual-domain constructs, Becker et al. described a triple-acting lytic enzyme that demonstrated potent activity against drug-resistant and intracellular *S. aureus* [62]. This study highlights how multi-catalytic fusion enzymes can overcome barriers such as intracellular persistence. Together, the studies described in this paragraph reinforce that dual or multidomain architectures, whether natural or engineered, are a cornerstone of next-generation therapeutic endolysins, and offer improved stability and potency with a wider antibacterial spectrum (Figure 5B).

### 4.4. Future Directions for Endolysin Catalytic-Site Engineering

Despite significant progress in endolysin catalytic-site engineering, several limitations remain that hinder the potential of endolysins to replace or supplement antibiotics. Many endolysins targeting Gram-positive bacteria present opportunities for optimizing specificity because their binding tends to rely on interactions with cell-wall components unique to their host organisms. Additionally, their catalytic functions often depend on CHAP and amidase activities, which exhibit sufficient variation to confer specificity at the species or even strain level. This restricts their applicability against Gram-negative pathogens. Although most studies have optimized the catalytic activity and specificity of engineered endolysins in phosphate-buffered saline or other buffer systems, the stabilities and immunogenicities of these endolysins under physiological conditions may differ. Consequently, endolysins can become ineffective, degrade, or be neutralized by antibodies in vivo, which reduces their therapeutic potential. Endolysin catalytic-site engineering is required for in vivo studies to ensure stability and functionality under physiological conditions. To date, no studies have reported resistance to endolysins, and it is considered rare compared with resistance to conventional antibiotics. However, the possibility of bacterial evasion necessitates continuous monitoring and the development of strategies to mitigate resistance. Furthermore, a deeper understanding and further documentation of bacterial peptidoglycan architecture are essential for optimizing catalytic activity. These knowledge gaps underscore the need for more systematic studies aimed at refining catalytic domains, substrate-access residues, and domain arrangements to enhance activity, broaden host range, and improve stability.

Emerging technologies offer promising avenues to overcome these challenges. Researchers have used directed evolution and rational protein engineering to improve the thermostability and lytic activity of enzymes like CD27L against *L. monocytogenes* [39,86]. AI-guided mutagenesis platforms, such as Pro-PRIME, allow for prediction of beneficial mutations to enhance the catalytic efficiency without exhaustive experimental screening [112]. PRIME also guides the evolution of protein stability, activity, and ligand affinity while enabling the development of mutants that function in harsh conditions such as extreme pH or high salt, which can be useful for designing mutations suited to solvents with different turgor pressures, such as Tris, PBS, and serum. Synthetic biology approaches exploit the modularity of endolysins, enabling the design of chimeric or multi-domain enzymes with improved potency, broader spectrum, and enhanced biofilm-disrupting ability, as reviewed by Gerstmans et al. [113]. Translating these advances into clinical applications will require scalable production methods, optimized pharmacokinetics for effective delivery, and rigorous regulatory evaluations to ensure safety and efficacy. Together, these strategies present a path forward for developing next-generation catalytic endolysins capable of addressing MDRO infections and biofilm-associated bacterial persistence.

## 5. Conclusions

Despite their recent arrival on the antimicrobial scene, endolysins are a promising tool to treat infections caused by MDRO. The number of endolysin candidates is limitless, especially with incorporation of metagenomic studies to identify more of these proteins [114]. As our understanding of these proteins increases, so does our ability to engineer them to enhance the antimicrobial effect. Our review explores the current paradigm of endolysin engineering, with the general theme involving library-based screening to filter the best candidates for cell-killing from a large variety of engineered endolysins. In combination with advances in AI, we expect exponential increases in available methods for generating suitable candidates to accelerate progress in the endolysin engineering avenues of peptide fusion, domain swapping, and catalytic site modification. These engineering avenues highlight the versatility of endolysin proteins as antimicrobial tools, which serve as a solid foundation for applying recent advances to maximize their antimicrobial potential. In all engineering cases, advances in structural biology will help us better understand the structure and function of endolysins, ultimately enhancing their use as antimicrobials. The future is bright for endolysin engineering research, and we look forward to further advances in this field.

## Figures and Tables

**Table 1:** Summary of recent advances in Artilysin engineering.

Approach	Example	Reference
Optimal endolysin-peptide linker length	LysPA26	[68]
In silico design pipeline	P362, P372	[69]
Endolysin-peptide combinations	Lys	[70]
Composite library screening	Dutarlysin-1,2,3	[71]

**Table 2:** Summary of recent advances in domain-swapping engineering.

Approach	Example	Reference
Large scale library screen	ClyC	[52]
Domain-swapping to treat bovine mastitis	Cly2v	[74]
Domain deletion	LysRODI	[75]
Domain-swapping bacteriophage proteins	ELP-E10	[76]

**Table 3:** Summary of endolysin catalytic site modifications.

Strategy	Approach	Key Examples	Main Modifications	Functional Outcome	Key Insight	Citations
Catalytic-site engineering	Target conserved peptidoglycan bonds	PlyC, LysK, Cpl-1, LysEF-P10, Exebacase, PlySs2	Selection/optimization of catalytic domains (CHAP, amidase, lysozyme)	Increased specificity and activity against MDR strains and biofilms	Catalytic-domain choice strongly dictates spectrum, efficiency and resistance profile	[1,2,3,4,5,6]
Dual catalytic-domains	Combine two domains	Cpl-711, PlySK1249, PlyC, LysSYL, Lys84, Lys109	CHAP/muramidase/amidase	Higher potency, lower resistance, biofilm disruption	Multiple bond cleavage improves robustness	[1,15,19,22,23,24,25]
Multi-catalytic domains	Combine multiple domains	Triple-acting lysins	Triple domain architectures	Active against drug-resistant/intracellular bacteria	Multi-domains overcome physiological barriers and persistence	[26]
Point mutagenesis	Single-residue substitutions	CD27L (L98 → W), T7 amidase (H37A), core variant (E88M) LysF1	Amino-acid changes in active, gating sites, or core.	Altered species specificity; increased activity and stability	Small mutations can tune specificity and activity	[7,8,9]
Charge engineering	Alter net charge of catalytic domain	Zn^2+^-dependent amidase; CHAP of PlyC	Electrostatic surface modification	Mixed or no improvement; sometimes reduced activity	Charge affects host range but is not reliable as a sole strategy	[10,11]
Metal-binding pocket engineering	Modify Zn^2+^/Ca^2+^ binding sites	Multiple Zn^2+^/Ca^2+^-dependent lysins	Mutations in metal coordination sites	Loss or reduction in activity	Metal-binding sites are poor targets compared to catalytic residues	[4,12,13,14]
Domain fusion	Swap/combine catalytic/binding domains	PlySK1249, LysK, Ecd18980CD	Rational fusion/truncation of EADs and CBDs	Enhanced lysis through domain synergy	Noncatalytic domains can enhance catalytic performance via positioning/synergy	[2,15,16]
Catalytic chimeras	Replace native EADs	Ply187AN-KSH3b, ClyH, Lys109	EAD replacement + CBD/domain fusion	Broader range (incl. MRSA), stronger activity	Domain swapping yields higher potency than parental enzymes	[17,18,19]
Endolysin–bacteriocin hybrids	Endolysin- bacteriocin domain fusions	SA.100, XZ.700, HY-133	Lysostaphin catalytic domain + SH3b domain + optimized linkers + endolysin catalytic domain	Increased activity and expanded range (incl. MRSA)	Linker and domain origin critically influence performance	[20,21]
